# VHL governs m6A modification and *PIK3R3* mRNA stability in clear cell renal cell carcinomas

**DOI:** 10.1172/JCI179560

**Published:** 2024-04-15

**Authors:** Hyemin Lee, Li Zhuang, Boyi Gan

**Affiliations:** 1 Department of Experimental Radiation Oncology, The University of Texas MD Anderson Cancer Center, Houston, Texas, USA.; 2 The University of Texas MD Anderson UTHealth Graduate School of Biomedical Sciences, Houston, Texas, USA.

## Abstract

N6-Methyladenosine (m6A), a prevalent posttranscriptional modification, plays an important role in cancer progression. Clear cell renal cell carcinoma (ccRCC) is chiefly associated with the loss of the von Hippel-Lindau (*VHL*) gene, encoding a component of the E3 ubiquitin ligase complex. In this issue of the *JCI*, Zhang and colleagues unveiled a function of VHL beyond its canonical role as an E3 ubiquitin ligase in regulating hypoxia-inducible factors (HIFs). It also governed m6A modification by orchestrating the assembly of m6A writer proteins METTL3 and METTL14, thereby stabilizing *PIK3R3* mRNA. Mechanistically, PIK3R3 contributed to p85 ubiquitination, which restrained PI3K/AKT signaling and consequently impeded ccRCC growth in cell and mouse models. This discovery provides potential treatment targets in *VHL*-deficient ccRCCs.

## Therapeutic challenges in ccRCCs

Clear cell renal cell carcinoma (ccRCC), comprising approximately 85% of primary renal cancers, is characterized by the loss of the von Hippel-Lindau (*VHL*) tumor suppressor gene (1). VHL functions as a component of the E3 ubiquitin ligase machinery, facilitating the degradation of the α-subunits of hypoxia-inducible factors (HIFs). HIFs are master transcription factors that orchestrate adaptive responses to hypoxia and consist of three α-subunits: HIF-1α, HIF-2α, and HIF-3α, among which HIF-2α is considered the most critical driver of ccRCCs (2, 3). In ccRCCs, the loss of *VHL* results in the stabilization of HIF2α, which acts as an oncoprotein, fueling the growth of renal tumors (4). Despite the clinical utilization of HIF2α-targeting drugs like PT2385 and PT2399, challenges persist due to inherent and acquired resistance (5, 6), underscoring the pressing need to identify additional mechanisms underlying VHL-mediated renal tumor suppression and develop therapeutic strategies for targeting *VHL*-deficient ccRCCs.

## m6A RNA modification in *VHL*-deficient ccRCCs

N6-methyladenosine (m6A) represents one of the most prevalent RNA chemical modifications in mRNA that substantially impacts RNA fate and expression by modulating cellular processes such as mRNA splicing, stability, nuclear export, and translation (7). In recent years, numerous studies have shed light on the crucial role of m6A in governing tumor proliferation, invasion, and metastasis, including in ccRCCs (8). Several investigations suggest that METTL14, a critical component of the m6A methyltransferase writer complex alongside METTL3 and WTAP, functions as a tumor suppressor in ccRCCs and highlight its role in attenuating the proliferation and migration capabilities of renal cancer cells (9, 10). However, the extent and specific mechanisms through which the m6A regulatory machinery is influenced by key oncogenic events in ccRCCs have remained elusive.

**Figure 1:**
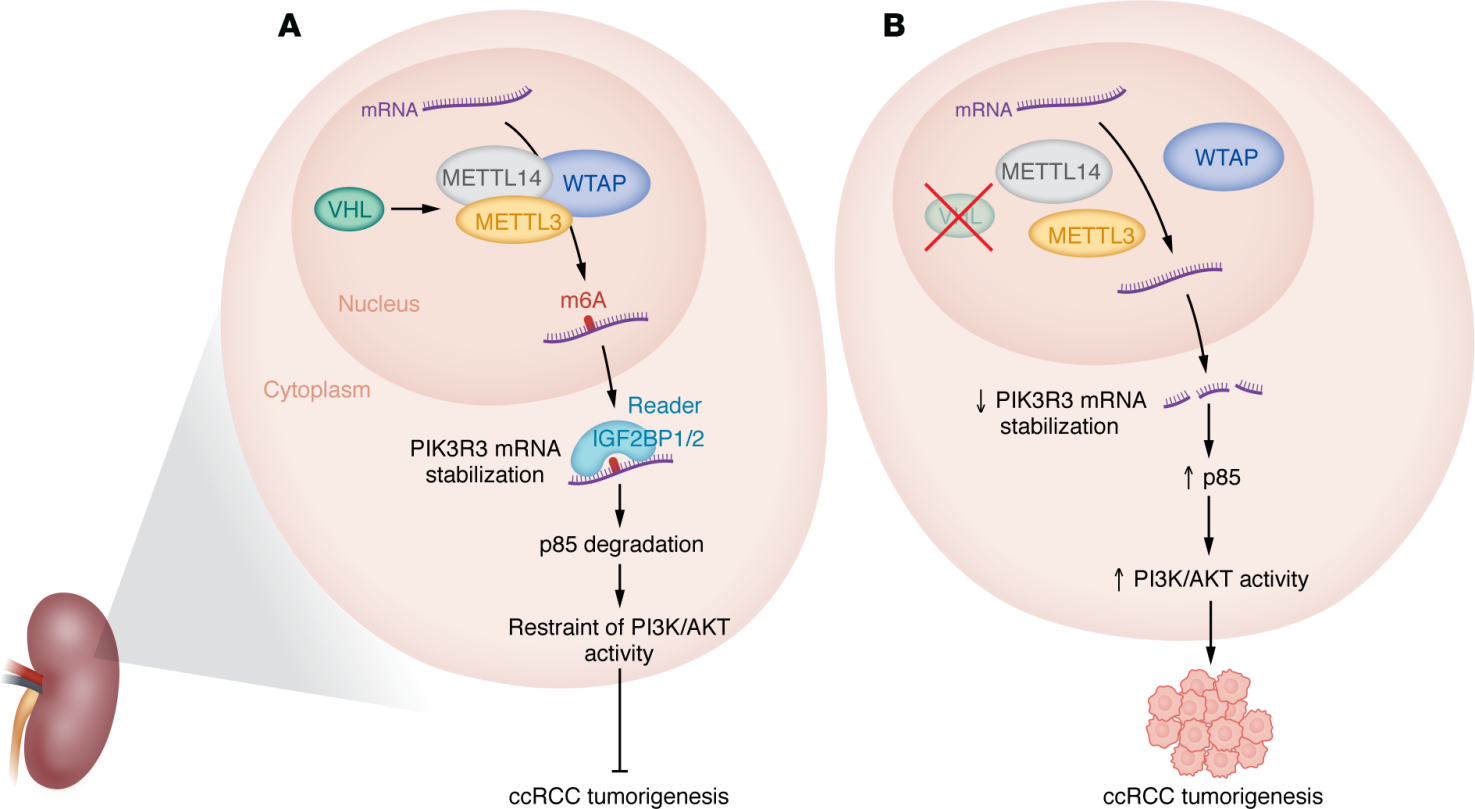
VHL mediates m6A RNA modification in ccRCCs. VHL orchestrates the complex assembly of METTL3 and METTL14, leading to the incorporation of m6A into *PIK3R3* mRNA. The m6A reader proteins, IGF2BP1 and IGF2BP2, play a role in stabilizing *PIK3R3* mRNA. The stabilized *PIK3R3* mRNA leads to increased levels of PIK3R3 protein, which in turn exert a negative regulatory effect on the p85 protein, consequently inhibiting PI3K/AKT signaling and impeding ccRCC tumorigenesis.

In this issue of the *JCI*, Zhang and colleagues have uncovered a role of VHL in regulating m6A modification and RNA stability in ccRCCs (11). Mechanistically, VHL interacted with proteins of the m6A enzymatic complex and modulated the interaction between METTL3 and METTL14, thereby regulating m6A levels in RCC cells (Figure 1). Depletion of VHL resulted in decreased global m6A levels in *VHL*-proficient renal cancer cells, with this regulation being mediated by VHL’s E3 ligase domain. Interestingly, VHL’s effect on m6A levels seems to operate independently of its canonical function in regulating HIFs. For example, the authors demonstrated that the overexpression of a HIF2α mutant that is resistant to VHL-mediated degradation in VHL-expressing RCC cells — in which the WT HIF2α protein is constitutively degraded by VHL — did not reverse the effect of VHL on m6A levels. In addition, contrary to VHL restoration, the deletion of *HIF2*α in RCC cells — which exhibit elevated HIF2α protein levels due to *VHL* loss — did not impact m6A levels.

Rigorous m6A RNA immunoprecipitation–sequencing (MeRIP-Seq) analyses revealed numerous differentially regulated m6A sites upon VHL depletion (11). In conjunction with RNA-Seq, Zhang et al. identified genes exhibiting differential m6A modification and expression changes upon VHL depletion. Notably, among these genes, the authors identified phosphoinositide-3-kinase regulatory subunit 3 (*PIK3R3*) as one of the implicated targets. Upon VHL depletion, the degradation rate of *PIK3R3* mRNA accelerated the decay process of *PIK3R3* mRNA. Conversely, overexpression of VHL decelerated its decay process. Consequently, VHL plays a critical role in maintaining the RNA stability of *PIK3R3*. Furthermore, IGF2BP1 and IGF2BP2 were identified as the primary m6A readers governing the regulation of *PIK3R3* mRNA. These studies suggest a model in which VHL facilitates the assembly of the METTL3 and METTL14 complex and increases m6A occupancy on *PIK3R3* mRNA, enhancing the stability of *PIK3R3* mRNA and consequently its protein levels (Figure 1).

Zhang and colleagues subsequently identified PIK3R3 as a regulator of the ubiquitination process involving the PI3K regulatory protein p85. Additionally, they demonstrated that p85α played a positive role in AKT activation (11), contrary to its function as a tumor suppressor in some other cancer types (12). *PIK3R3* mRNA stabilization by VHL through m6A modification resulted in elevated protein levels of PIK3R3, which then negatively regulated p85 and attenuated PI3K/AKT activation, subsequently impeding renal tumor growth. Conversely, *VHL* loss in ccRCC cells decreased PIK3R3 levels, resulting in AKT hyperactivation and enhanced renal tumor growth (Figure 1).

## Conclusions and future perspectives

Zhang et al. revealed a role of VHL protein in the regulation of m6A modification and *PIK3R3* mRNA stability in ccRCCs (11). Beyond its canonical function as an E3 ligase in the regulation of HIF protein stability, VHL’s E3 ligase domain mediates the interaction with METTL3 and METTL14, serving as an adaptor to facilitate the assembly of the METTL3/METTL14 complex. Notably, this regulatory process does not appear to induce alterations in the expression levels of METTL3 and METTL14 and operates independently of its canonical substrates such as HIF1α and HIF2α.

This compelling study also prompts several intriguing questions for future research endeavors (11). Firstly, there is a need for deeper investigation into the mechanisms through which VHL regulates m6A and PIK3R3 levels in an E3 ligase–dependent manner while not impacting METTL3 protein stability. Further understanding this regulatory mechanism could identify therapeutic targets for combating *VHL*-deficient ccRCCs. Moreover, considering the prominent role of HIF2α in driving *VHL*-deficient renal tumor growth, it remains critical to elucidate the contribution of PI3K-AKT hyperactivation in mediating renal tumor growth in *VHL*-deficient ccRCCs. Exploring the crosstalk between these signaling pathways could uncover synergistic therapeutic strategies by coinhibition of HIF2α and PI3K/AKT pathways for managing *VHL*-deficient renal tumors.

The dysregulation of m6A RNA modification and its associated machinery, encompassing writers, readers, and erasers, is a recurrent observation across various cancer types. Numerous studies have investigated the role of m6A in diverse pathways, leveraging the modulation of m6A as a targeted therapeutic approach tailored to specific cancer subtypes, given its dual function as both an oncogene and a tumor suppressor. The efficacy of combining inhibitors or agonists targeting m6A regulators with other therapeutic modalities has been demonstrated in previous studies (13), underscoring the potential of this approach in cancer treatment. Additionally, besides exploring m6A writer proteins, there lies an opportunity to investigate whether VHL also influences m6A readers or erasers, thereby further unraveling the interplay between VHL and m6A machinery. Importantly, recent findings have identified the m6A demethylase FTO as a synthetic lethal partner of VHL in ccRCCs (14), suggesting the potential therapeutic relevance of targeting FTO in *VHL*-deficient ccRCCs. Therefore, further exploration of FTO inhibitors in *VHL*-deficient ccRCCs, coupled with an in-depth investigation into the clinical implications of m6A-mediated regulation, holds promise for the development of therapeutic interventions tailored to *VHL*-deficient ccRCCs.

## Version 1 04/15/2024

Electronic publication
